# Pilot of a multicomponent program for people with dementia and their care partners: Health services staff expectations, experiences and observations

**DOI:** 10.1177/14713012251315527

**Published:** 2025-01-15

**Authors:** Nathan M D’Cunha, Georgina Chelberg, Helen Holloway, Lara Wiseman, Angie Fearon, Jane Kellett, Stephen Isbel, Kasia Bail, Ian Huang, Diane Gibson

**Affiliations:** School of Exercise and Rehabilitation Sciences, Faculty of Health, 2234 University of Canberra, Bruce, ACT, Australia; Centre for Ageing Research and Translation, 2234 University of Canberra, Bruce, ACT, Australia; Centre for Ageing Research and Translation, 2234 University of Canberra, Bruce, ACT, Australia; School of Nursing, Midwifery and Public Health, Faculty of Health, 2234 University of Canberra, Bruce, ACT, Australia; School of Exercise and Rehabilitation Sciences, Faculty of Health, 2234 University of Canberra, Bruce, ACT, Australia; School of Exercise and Rehabilitation Sciences, Faculty of Health, 2234 University of Canberra, Bruce, ACT, Australia; Centre for Ageing Research and Translation, 2234 University of Canberra, Bruce, ACT, Australia

**Keywords:** dementia, rehabilitation, allied health, multicomponent, multidisciplinary, caregiving, care partner, cognitive stimulation therapy, post-diagnostic

## Abstract

There is increased recognition of the need to improve post-diagnostic pathways for people with dementia and their care partners living in the community to access rehabilitation services to support independence and wellbeing. However, there is minimal evidence on implementing rehabilitation services for this population. The study aimed to present the expectations and experiences of allied health staff involved in piloting the Sustainable Personalised Interventions for Cognition, Care and Engagement (SPICE) program based at an outpatient clinic of a public rehabilitation hospital. Over twelve weeks, the program combines small group and dyadic evidence-based interventions including cognitive stimulation therapy, occupational therapy, physical activity, care partner education, and dietetics. A qualitative exploratory methodology was used with pre- and post-program interviews conducted with ten allied health staff. Questions were designed to elicit the expected and actual benefits and challenges of the initial implementation of the multicomponent program. The multidisciplinary team were motivated by the potential for the SPICE program to meet an important service gap, and confident people with dementia and their care partners would benefit. Staff reported enjoyment, satisfaction, and confidence in delivering the program, and believed the multiple components had synergistic effects on participants, particularly regarding social connection and functional engagement. While staff had pre-program concerns regarding retention, participant fatigue, and managing challenging situations, these were not realised. At times, staff observed program intensity to cause unintended stress for some care partners. Resourcing and strategies to ensure sustainability were identified as important by staff, as well as the need for ongoing support to participants post-program. Overall, the SPICE program exceeded expectations and was rewarding for staff. Further work to refine and evaluate the program is necessary to support its potential to continue providing dementia rehabilitation to promote the independence and wellbeing of people with dementia and care partners living in the community.

## Introduction

Growing evidence has demonstrated the value of collaborative, comprehensive, person-centred rehabilitation interventions for people with dementia and their care partners following a diagnosis of dementia. Effective multicomponent programs can include social engagement through group activities, exercise, psychoeducation, and a variety of allied health supports (Laver, Cumming, et al., 2016; Laver, Dyer, et al., 2016; Livingston et al., 2024). For most people with dementia, post-diagnostic interventions have potential to maintain independence, wellbeing, and participation in activities of daily living (O’Connor, Gresham, Poulos, Hudson, et al., 2020), improve quality of life (Kishita et al., 2019; McDermott et al., 2019), delay entry to residential aged care (Eaglestone et al., 2023), and improve relationship quality with family members or friends (Gitlin et al., 2010; Laver, Dyer, et al., 2016). Coordinated services for people with dementia living in the community have been shown to reduce neuropsychiatric symptoms and care partner burden (Backhouse et al., 2017).

Care partners of people with dementia also benefit from post-diagnostic interventions through information and resources, training, and access to respite services so they can develop self-advocacy and caregiving skills (Livingston et al., 2024). Additional benefits can include reduced emotional stress, improved coping skills, enhanced quality of life and maintenance of wellbeing (Casey et al., 2024; Gitlin et al., 2010; Laver, Dyer et al., 2016). This is significant, given the intensity of providing upwards of 60 hours per week of care to a person with dementia in the community (Australian Institute of Health and Welfare, 2023). A scoping review of interventions targeting the relationship between people with dementia and spousal care partners concluded the quality of evidence for benefits was poor due to a combination of inadequacies in study design, outcome measures and theoretical underpinnings for the various diverse interventions (Gilbert et al., 2023). However, only one study in this review employed a multicomponent intervention (Chung, 2001).

While evidence concerning potential benefits to people with dementia and their care partners is strengthening, access to services remains poor, and multidisciplinary implementation of post-diagnostic programs has proven challenging (Cations et al., 2019). There is an ongoing need to improve pathways for people with dementia and their care partners to access rehabilitation interventions, and the evidence-base is growing and recognised as part of the World Health Organization’s Rehabilitation call for action (Jeon et al., 2023). Access to post-diagnostic dementia services commonly requires individuals to seek out services independently or may, in some instances, occur following an acute hospital admission (Kable et al., 2015; Pavković et al., 2023). While memory clinics in Australia provide important multidisciplinary services such as receiving and communicating a dementia diagnosis, medication management, and referrals to community services, their capacity to provide post-diagnostic intervention is currently limited (Low et al., 2023; Pavković et al., 2023).

A wider national, societal, multi-stakeholder health and social care system approach is warranted to enable people with dementia to ‘live well’ and their care partners to receive appropriate recognition and support (Gauthier et al., 2022; Quinn et al., 2022; World Health Organization, 2017). Rehabilitation interventions have the potential to reduce the economic and social impacts of dementia through reduction in longer term healthcare services and unnecessary hospitalisations (Kjerstad & Tuntland, 2016; Rahja et al., 2020). Allied health professionals have a key role in enabling access to appropriate support (Quinn et al., 2022) and designing and delivering appropriate interventions. Allied health professionals’ unique skill sets enable tailored interventions that meet the individual needs, abilities, mobility, and preferences of the person with dementia and their care partner. These positive impacts have been observed in a novel multicomponent, therapeutic, post-diagnostic rehabilitation intervention for people with dementia and care partners known as the Sustainable Personalised Interventions for Cognition, Care and Engagement (SPICE) program (D’Cunha et al., 2023).

Better understanding of the ‘on the ground’ and organisational challenges and benefits experienced by staff contributes to the Australian National Dementia Action Plan’s broad vision of enhancing dementia research, innovation, and developing more effective, person-centred care strategies for the growing number of Australians affected by dementia (Department of Health and Aged Care, 2024). The SPICE program is a comprehensive post-diagnostic rehabilitation intervention which combines evidence-based interventions for people with mild and moderate dementia and their care partners. By providing group-based and personalised support, the program addresses key national priorities in the Australian National Dementia Action Plan 2024-2034 by aiming to slow the progression of dementia, enhance quality of life, help maintain independence in the community, and reduce avoidable hospitalisations (Department of Health and Aged Care, 2024). The SPICE program is unique in its multidisciplinary approach within an outpatient rehabilitation clinic in a public hospital and its pilot implementation is relevant to other services designing new dementia rehabilitation services.

The aim of this study was to present the expectations, experiences, and observations of health services staff involved in the initial implementation of the SPICE program before commencement in 2022 and after the first group was completed. This study documents staff perspectives on the challenges and successful aspects, and suggested modifications for ongoing implementation of an innovative multicomponent program within a conventional rehabilitation hospital setting.

## Methods

### Study design

This study employed a qualitative exploratory methodology to explore the expectations and experiences of the health services staff involved in delivering the initial implementation of the SPICE program. Semi-structured interviews were conducted pre- and post-program. This research design provides insights into the experiences and challenges of piloting a new service in a public rehabilitation hospital that had not previously delivered any dementia-specific services. The study was approved by the University of Canberra Human Ethics Research Committee (#9301).

### Setting and context

The study was based at the University of Canberra Hospital Specialist Centre for Rehabilitation, Recovery and Research. The hospital is a public health purpose-built rehabilitation centre in the Australian Capital Territory. The SPICE program aims to address a gap in dementia-specific services in the Australian Capital Territory for people with dementia and their care partners. Canberra Health Services approached University of Canberra researchers to collaboratively design, develop, implement, and evaluate a multidisciplinary, multicomponent post-diagnostic intervention drawing upon best available evidence, with additional consultation with Dementia Australia Advocates.

**Figure 1.**
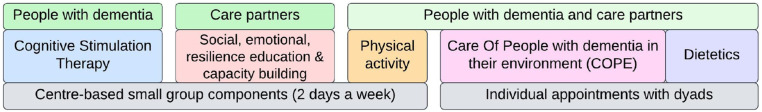
Weekly structure of the 12-week multicomponent SPICE program components.

The SPICE program and inclusion criteria have been previously described (D’Cunha et al., 2023). In brief, the SPICE program is a twelve-week multicomponent, post-diagnostic rehabilitation program for people with dementia and care partners. Up to seven dyads (seven people with dementia and their seven care partners) participate in each group. The SPICE program aims to delay progression of dementia and improve quality of life through participation in a combination of evidence-based allied health psychosocial and rehabilitation interventions completed in small groups and as a caregiving dyad: 1) Cognitive Stimulation Therapy (Desai et al., 2024); 2) Carer Social, Emotional, and Resilience Education and Capacity Building (Parkinson et al., 2017; Pierse et al., 2021); 3) Physical activity; 4) Care of People with dementia in their Environments (COPE) program (Clemson et al., 2021); and 5) dietary assessment and advice (Figure 1). The SPICE program is primarily delivered by allied health professionals at Canberra Health Services, with University of Canberra clinical academics providing two of the twelve workshops to care partners and the dietary assessment and advice component. Dementia Australia provide an information and question and answer workshop in the final week of the program.

The SPICE program was originally designed to be a pilot program to evaluate whether the combination of interventions worked, whether it was feasible, and to evaluate potential benefits people with dementia and care partners receive. While Canberra Health Services provides a number of services that can be accessed by people with dementia, particularly following a hospitalisation, there were no dementia-specific services incorporating dementia rehabilitation and reablement or care partner support. The unique design of the SPICE program involves a mix of small group and dyadic-based components to enhance quality of life. This combination may have synergistic effects, which will be explored in future work as the program evolves.

### Data sources

The allied health team delivering the SPICE program includes professionals from occupational therapy, neuropsychology, clinical psychology, physiotherapy, social work, speech pathology, dietetics, and allied health assistants. All staff involved in the development and delivery of the program were invited to an interview before the first group commenced. Inclusion of all staff was intended to capture a broad range of views from the multidisciplinary team as well as team members with varying levels of experience working with people with dementia and care partners. As the SPICE program is a multicomponent intervention, different perspectives were anticipated based on the components each team member was involved in. Consent was requested from staff by email and then online or in-person interviews were conducted depending on COVID-19 mitigation procedures in place at the time of interviews. Only staff involved in delivering the SPICE program were invited to participate in the follow-up interviews.

### Data collection and analysis

Interviews were conducted prior to program commencement and after the first SPICE program group completed the multicomponent intervention in December 2022. Semi-structured interviews with open-ended questions were used to explore the expectations and experiences of the allied health professionals to explore contextual factors regarding running the first SPICE program, but with scope for interviewers to explore ideas and concepts which arose during the interviews (Adeoye-Olatunde & Olenik, 2021). Questions were designed to elicit information about the background of the staff member and their prior experience working with people with dementia, their role in the development and implementation of the SPICE program (pre-program only), expectations on what aspects of the program would be beneficial or challenging, and how they felt the people with dementia and care partners would find the program (See Supplemental Figure 1). In the post-program interviews, questions on their experiences followed a similar pattern. However, staff were also prompted to respond to their views from their pre-program interviews and to describe a memorable event from their delivery of the program.

Audio recordings from all interviews were transcribed verbatim, and staff were provided with the opportunity to review the transcripts before analysis to ensure accuracy and trustworthiness. Data were read and coded by five authors using content analysis (Graneheim & Lundman, 2004). This process involved repeatedly reading the text to interpret and understand the context before assigning meaning units. Meaning units were coded based on their content, then compared and sorted into subcategories and then categories, based on their similarities. Two authors (G.C & D.G) consolidated and condensed data into themes using an iterative process before being reviewed and discussed with a third author (N.D) to triangulate and refine the findings.

## Findings

Nine health services staff completed pre-pilot interviews, and eight completed post-pilot interviews. Interviewees included a services manager, allied health professionals and allied health assistants. The allied health assistant was assigned to the program without enough time to conduct an interview but was interviewed post-program. Additional demographics of staff are presented in Table 1, including years of experience, specialty, experience working with people with dementia, and role during the SPICE program.

**Table 1.** Staff background information.

	SPICE role/s	Experience (years)	Experience working with older people	Experience with people with dementia and/or care partners	Experience working with dementia-specific services	Pre-program interview	Post-program interview
Allied health assistant	CST, physical activity	0.5	Yes	Yes	No	No	Yes
Dietitian	Dietetic consultations	14	Yes	Yes	Yes	Yes	Yes
Clinical psychologist	Care partner education	29	Yes	Yes	Yes	Yes	Yes
Physiotherapist	Physical activity	4	Yes	Yes	Yes	Yes	Yes
Occupational therapist 1	COPE	35	Yes	Yes	No	Yes	No
Occupational therapist 2	COPE, CST	3	Yes	Yes	No	Yes	Yes
Neuropsychologist 1	CST	7	Yes	Yes	Yes	Yes	No
Neuropsychologist 2	CST	4	Yes	Yes	Yes	Yes	Yes
Services manager	Coordinator	25	Yes	Yes	Yes	Yes	Yes
Social worker	Care partner education	13	Yes	Yes	Yes	Yes	Yes

Key: COPE: Core of People living with dementia in their environment; CST: Cognitive stimulation therapy.

Content analysis of the interviews followed the established line of enquiry and presented within three content areas (A) Benefits of the SPICE program; (B) Challenges of the SPICE program; and (C) Suggestions to improve the SPICE program. The identified themes are presented as ‘Expectations’ (pre-program) and ‘Experiences & Observations’ (post-program) and listed in Table 2. Findings are described as follows, accompanied by exemplary quotes, with further notable quotes indicated in brackets and listed in Supplemental Materials 1 (Tables A-C).

**Table 2.** Content areas and themes.

Content areas	Themes pre-intervention	Themes post-intervention
Benefits of the SPICE program	Addressing a service gap	Addressing a service gap
	Benefits for staff	Benefits for staff
	Benefits for care partners	Benefits for care partners
	Benefits for people with dementia	Benefits for people with dementia
	Importance of program evaluation	
		Social connection and relationships
Challenges of the SPICE program	Participant recruitment and retention	Participant recruitment and retention
	Appropriate resources	Appropriate resources
	Staff capability to respond to challenging situations	Staff capability to respond to challenging situations
	Sustainability of the program	Sustainability of the program
Suggestions to improve the SPICE program	Ongoing support as a follow-up to the SPICE program	Ongoing support as a follow-up to the SPICE program
	Enhanced cultural inclusivity	
		More individualised advice during the program
		Clearer communication with care partners
		Consideration of the program intensity

### Benefits of the SPICE Program

Prior to the intervention, the staff involved were positive about the potential of the program. Key themes were (a) addressing a service gap; (b) benefits for staff; (c) benefits for care partners; and (d) benefits for people with dementia. Staff also emphasised the (e) importance of program evaluation. Following the intervention, staff reflected on its successful realisation and re-stated themes of meeting a service gap, benefits to staff, people with dementia and care partners. An additional theme of (f) social connection and relationships was identified post-intervention.

#### Addressing a service gap

Prior to the program commencing, staff emphasised the SPICE program would fill a service gap in the ACT community, providing ‘a path forward’ rather than just wishing someone ‘good luck’ (A1). They also valued the breadth of the intervention across cognition, physical health, and quality of life (A1-A3) and were aware of its innovative nature, described by one staff member as ‘unprecedented in public health’ (A1).

“‘I think it's excellent. And I think it's really needed and given that I am often the one giving the bad news to people with dementia, the idea of having a path forward rather than just going: ‘There's your label. Sorry about it. Good luck.’ Yeah, that, that feels really nice…like it’s the amount of services provided through this program - is kind of unprecedented in public health.”’ (A1)

After the intervention, staff affirmed the value of the SPICE program as an innovative multicomponent program (A22-A25). However, there was a much stronger awareness of, and emphasis on the synergy of the multiple domains that made the program a success: ‘all the features of the group of the whole program like the cognitive stimulation therapy and the carer wellbeing and the exercise and everything, all worked really well together’ (A23). Examples of specific elements that worked together included the interaction between the COPE home visits and dietetics sessions (A22) and how information gleaned in the COPE visits helped support cognitive stimulation therapy (A25). Staff also reflected on program quality, achieved through teamwork and insights of the allied health team. This expertise was applied through participant interactions and tailored activities to suit a variety of needs and abilities for engagement in the SPICE program sessions (A24).

#### Benefits for staff

Prior to the intervention, staff spoke of the individual and collective motivation to provide dementia rehabilitation and the potential rewards this would bring for staff (A4-A6).

“Most of the clinicians who were keen to be involved have probably experienced the level of distress and despair...of a care support provider, you know, or somebody living with dementia…that’s probably what I think is one of the things that drives people.” (A4)

The SPICE program was viewed as a valuable professional opportunity to collaboratively deliver a multidisciplinary program (A6).

Staff were delighted about the post-program changes for participants’ communication and the rapport developed amongst the group, describing the experience as ‘fantastic’ (A26) and ‘a great highlight’ (A27). Professional opportunities included increased dementia knowledge and interdisciplinary learning as staff co-delivered sessions. For example, while the allied health assistant had experience working with older people, they had not been involved in a program specifically for people with dementia or their care partners. In addition, the program provided supervised learning opportunities, which were rewarding to staff (A28).

#### Benefits for care partners

Health services staff expected that the structure of the SPICE program, with formal and informal sessions, would be a valuable experience for care partners (A7) and supporting them would be ‘one of the key things’ (A8). Support and practical caregiving strategies would help manage changed behaviour, reduce stress, improve quality of life, and potentially help their loved one with dementia to remain at home (A8-A10).

After program completion, staff comments aligned closely with their expectations concerning the value of engagement approaches and individualised strategies for care partners. This provided ‘a better understanding of how to…engage them and how to support them, and like how to pitch it at the right level’ (A29-30). There was an emphasis on manageable changes suited to the individual and the importance of empathy (A31), recognising the complex and significant role of dementia caregiving and the importance of ‘self-compassion’ (A30-32). Staff expected to positively impact quality of life for care partners and people with dementia (A33).

“…watching the growth of the patients and their carers. And just hearing time and time again, the feedback and the positive feedback and the impact that it's made to the health, wellbeing, and quality of life of both groups of people…has been…really the highlight of the year from a work perspective.” (A33)

Care partners also offered to provide written or verbal support to help others like them have the same opportunity (A34).

#### Benefits for people with dementia

Staff acknowledged the potential benefits of the SPICE program and expressed a ‘belief’ in rehabilitation for people with dementia as an ‘underlying motivator’ of their work (A11). They referred to the significance of the ‘holistic’ and ‘cohesive’ effect of the different components of dementia care, commonly experienced in standard clinical practice as ‘bits and pieces’ (A12-13). The multidisciplinary approach of the SPICE program was expected to lead to improvements in quality of life and functional engagement for people with dementia, driven by gains in different dimensions of health (A14-15).

“…that kind of functional engagement in the world. [In a previous cognitive stimulation therapy pilot] we saw that increase a fair bit…well, their cognition on screening went up, which I wasn't really expecting…I think their physical fitness will probably improve if they are not getting a lot of exercise currently. As I said, I don't know a lot about COPE, but I would think having tailored strategies in the home would also be really, really beneficial. In terms of getting people a bit more engaged in just everyday life, and not sort of sitting off in a corner isolated from things.” (A16)

Staff emphasised the evidence-base for the components gave them confidence there would be positive outcomes including physical activity for strength, mobility, and capacity (A15); cognitive stimulation therapy for cognitive and social engagement (A17); and COPE for tailored meaningful occupation, safety, and delay of functional decline (A18). Several staff also thought participants would find aspects of the program ‘fun, supportive and enjoyable’ (A19).

Post-intervention, staff comments resonated with their expectations about the positive cognitive, social, and physical outcomes for people with dementia. The regularity of sessions over the twelve weeks allowed people with dementia to become familiar with the routine and understand what was expected of them - described by staff as ‘carry over’ (A35). For example, the physiotherapist found the repetitive exercises enabled participants to progress around the stations more independently. While the first couple of sessions were ‘such hard work,’ in the last half of the program ‘we were just there telling them how great they were’ (A36). Staff also commented on the gains in quality of life (A33), and increased motivation and engagement inside and outside of the SPICE program (A37). Furthermore, staff observed enhanced social confidence, with people who were initially reserved and/or experiencing word-finding challenges starting to initiate conversations and interactions (A38). This echoed the staff’s expected improvements in functional engagement (A16). For example, the service manager shared the significant changes they observed in a participant who, at the beginning, ‘hardly spoke a word’ but, one month into the program, brought in their Australian rules football and initiated a mini-coaching session to teach them how to bounce the ball. In her words, ‘It was awesome’ (A39).

#### Importance of program evaluation

Given its potential benefits, the sustainability of the SPICE program was an important consideration for the staff prior to commencement, and they emphasised the need for an evidence base to secure future funding (A20). Another staff member saw evaluation as a way of demonstrating that people with dementia were legitimate recipients of rehabilitation services: ‘if we're looking at rehabbing, offering people the means to have the best quality of life that you can have, then certainly anybody's entitled to that’ (A21).

#### Social connection and relationships

While there was reference to social aspects of the SPICE program pre-intervention, it was strongly acknowledged in post-intervention discussions. Social connections developed between people with dementia, care partners, and staff over the twelve-week intervention. Staff felt these connections helped ‘create a cohesive group [who] really supported and cared for one another’ (A40). They also described relationship benefits for dyads – bringing ‘fun back into their relationship’ (A41) and ‘something for us to do together’ (A42). Staff spoke positively about the developing friendships they observed between care partners (A43) and between people with dementia (A44). Both types of social connections were a valuable source of emotional support and camaraderie during the program, and care partners initiated their own online chat group to stay connected after the program’s conclusion (A43-45).

Several staff reflected on the memorable interactions they had experienced with participants, with one recalling the sincere gratitude and validation expressed by a person with dementia regarding the respect and recognition shown to them by staff throughout the SPICE program (A46).

### Challenges of the SPICE program

Prior to the intervention staff involved in program development and implementation expected the main challenges to be (a) participant recruitment and retention; (b) appropriate resources; and (c) staff capability to respond to challenging situations. They also expressed concern regarding (d) sustainability of the program. Post-intervention, staff returned to the same topics but with varying degrees of concern.

#### Participant recruitment and retention

Prior to the intervention, staff were concerned it would not prove feasible to recruit a sufficient number of participants who were able and willing to commit to the length and intensity of the program. This included concerns about physical and cognitive fatigue for people with dementia over twelve weeks, particularly given the multiple program components. One staff member described the program as ‘busy’ (B1), and another felt the length could be ‘challenging’ and had concerns about fatigue and illness leading to attrition (B2).

“I do think the program is busy. There are lots of components. And I, whilst I'm excited that we can put all of these together and provide that for people, I am a little bit worried about overload.” (B1)

In practice, participant recruitment and retention did not prove to be a challenge, and the people with dementia did not leave on account of fatigue, health issues or the length and intensity of the program. This was somewhat of a surprise to staff (B10).

“…it totally flipped on its head my expectation that if it's too long, it's too long for people to engage with, and clearly, they didn't think it was long enough. So, it was just so different to our usual rehab cohort, not attending appointments and so forth. So that was a surprise for me.” (B10)

However, staff reported the program structure was, at times, overwhelming for the care partners. This was primarily due to the logistics of attending two in-person sessions on-site per week, as well as regular COPE home visits. Some care partners reported to staff a sense of stress and guilt for not having trialed new strategies they had learnt during COPE sessions before the next session where they were ideally supposed to share whether it worked in practice (B11-12).

#### Appropriate resources

As a new program being introduced without additional funding, staff expressed concerns that resources would be taken away from existing clinical services (B3), with one example being the time commitment from the small neuropsychology team (B4). Staff also pointed out that as the service manager had played a key role in championing and implementing the SPICE program, quality and continuity could be compromised if that person fell ill or left their role (B5-6).

“You do need a champion; you need a champion who is not going anywhere for a little bit. Because there is actually quite a high turnover in a lot of allied health.” (B6).

Post-intervention, staff reflected on how they could balance their role in the SPICE program with overall program planning to ensure they completed their regular workloads, including competing commitments like mandatory training (B13). Meeting organisational requirements such as training, consistent documentation, and preparation time were considered challenges going forward. The service manager gave similar feedback, however, also indicated there was potential for efficiencies in terms of lessons learned.

“I think now…it doesn't feel like the time investment was as great as it felt like it was going to be on paper. So, for example, I think we put on one OT this time, because we, we wanted to make sure that they were able to establish a new program. But we now feel like we would only need an additional half an OT to run two programs…I think, you know, after you run the first group, you get a little bit, you find your efficiencies.” (B14)

#### Staff capability to respond to challenging situations

Pre-intervention, there was some uncertainty about the capacity of the team during incidents of changed behaviour where people ‘don’t feel like they’ve got the skills and knowledge to manage’ or in the event of a fall, or an episode of intense emotions by a care partner or a person with dementia (B7). Another potentially challenging aspect of delivering the group sessions included personality clashes or behaviours that could distress others, including personal disinhibition (B8).

There were, in fact, no episodes beyond staff capabilities. In the words of one staff member: ‘it didn't end up being the most challenging thing’ (B15). There were some challenges in facilitating group education sessions that involved a lot of important content, such as when some care partners showed clear signs of distress. They felt a competing need to validate people’s emotions and manage group learning (B16). At times, it was difficult for everyone in a facilitated group session to have a voice when there was a dominant participant (B17).

#### Sustainability of the program

Staff were pragmatic about the need to obtain significant and influential results to obtain ongoing funding for the SPICE program. While they expected meaningful outcomes for participants, communicating this effectively to those in a funding environment was perceived as challenging for a program of this kind. A staff member described this very clearly:

“Being able to identify the value that comes from that program and being able to compete in a financial market where you can demonstrate the benefit…in line with, you know, the drivers for most health services, which are bed days.” (B9)

After the intervention, staff remained concerned about the long-term sustainability given the staff resourcing costs. However, they could see approaches that would offer efficiencies of scale, such as running two groups in parallel (B18). Additionally, some participants had told staff they would pay for the SPICE program, which was not only a program opportunity but signified how much they valued the program (B19).

### Suggestions to improve the SPICE program

Prior to the intervention, staff had two main suggestions that should be considered for future iterations. These were arranging (a) ongoing support as a follow-up to the SPICE program; and (b) enhanced cultural inclusivity. Post-intervention, staff re-iterated their original views concerning the need for ongoing support once participants had completed the program. Three additional sub-themes were identified as potential inclusions: (c) more individualised advice during the program; (d) clearer communication with care partners; and (e) consideration of the program intensity.

#### Ongoing support as a follow-up to the SPICE program

Prior to the intervention, both the management and allied health staff could see valuable additions that would be limited by resource implications. For example, having ‘some regular check-ins and support access after the program ends’ would be ideal for participants. Furthermore, if complex issues were raised in group sessions or COPE visits, staff would need an appropriate way of following up (C1-2). Ongoing support would prevent participants from feeling a program ‘drop off/disconnect’ where they returned to a weekly routine with minimal dementia support.

Post-intervention, staff retained this view and were firmer and more specific in expressing this point (C5-C7). For example, one staff member said of care partners ‘they just got so much value out of the peer support and of that regular, structured engagement with other people, and I think it is really difficult just for that to stop’ (C6). Another specifically mentioned the lack of options for continuing cognitive stimulation therapy (C5). The current lack of capacity to provide these services was a missed opportunity to maximise and prolong the benefits observed in the program.

#### Enhanced cultural inclusivity

Staff were aware prior to program commencement that the SPICE program’s current format may not be culturally inclusive, particularly for those for whom English was their second language (C3). No comments were made on this point post-intervention.

#### More individualised advice during the program

Staff reported requests from participants for referrals to a speech pathologist and social worker and additional individual advice, as the group setting did not always allow for conversations about sensitive care challenges. Staff recognised the diverse health and social needs across the group and some aspects of dementia care were not practical for a group setting (C8-9). For example, physiotherapy assessment and advice about future mobility needs were not possible, so only general advice could be provided (C10). Optional sessions for care partners could be a valuable addition to the program, and the need for referrals for more nuanced care challenges was identified.

#### Clearer communication with care partners

Building on the feedback about the inability to offer individual support, several staff felt future programs should involve early and clear communication with care partners. Staff envisioned this as an opportunity to establish expectations about the aims and scope of the program, and what care partners wanted out of the SPICE program, particularly in relation to specific and complex participant medical or social needs (C11-12).

#### Consideration of the program intensity

As noted earlier, expected challenges for people with dementia in dealing with program intensity did not arise, however staff discussed some concerns expressed by care partners as to their capacity to implement all that they were learning while managing the SPICE sessions and other commitments. In response, several staff shared ideas about changing the program structure, such as staggering the SPICE schedule, so COPE commenced before the cognitive stimulation therapy sessions or lengthening the program. This would enable care partners to spread the time commitment and enhance staff awareness of the needs of individual dyads (C13-14).

## Discussion

This paper presents the views of staff who implemented an innovative multicomponent intervention for people with dementia and their care partners designed to address an identified gap in dementia-specific service delivery. There was consistent support for the SPICE program amongst staff and high readiness to implement the program, and it is not surprising they had positive expectations. As most were experienced allied health professionals, it is also not surprising they were aware of likely challenges and ideas for potential improvements. Working across three broad streams set out in the findings, it is possible to identify matters related to the service level, the program itself, and the staff.

At the service level, the SPICE program was developed as a collaboration between allied health and researchers meaning that staff buy-in was part of the program’s identity from the outset. The major staff concerns of program resourcing and sustainability are common when implementing post-diagnostic dementia care (NHMRC National Institute for Dementia Research Special Interest Group in Rehabilitation Dementia, 2021; Wheatley et al., 2021). As the pilot program was being implemented without additional resources, there was concern about the potential negative impact on staff capacity for existing services. While the reality of staff resourcing impacts were deemed to be less than expected, concern about securing ongoing resourcing beyond the pilot remained evident. Program evaluation data demonstrating the positive impact of the program with the potential for additional funds were seen as the best pathway to sustainability. This is consistent with previous findings among clinicians in Australia (Cations et al., 2020). Other barriers to implementing post-diagnostic dementia services include an unsupportive infrastructure, and limited capacity and capability with health services (Wheatley et al., 2021). However, creative use of funding and strategic alliances are key strategies for successful implementation (Wheatley et al., 2021). In the present study, the collaborative work between allied health and researchers was seen as a key to piloting the program.

In relation to the SPICE program itself, staff identified its unique value as a multicomponent program delivered collaboratively by allied health staff – facilitating a holistic approach to post-diagnostic care. They were positive about the opportunity to work in this innovative and multidisciplinary way and affirmed the evidenced-based components would facilitate positive impacts. Their major concerns around the intensity of the program impacting recruitment and retention did not eventuate. However, staff observed some unintended stress for care partners engaging in up to ten COPE sessions at home in conjunction with attending and engaging with the bi-weekly sessions. As COPE is typically provided over four months (Clemson et al., 2021), staff considered whether participants could complete this following the on-site group components. This would extend the intervention, and potentially provide greater benefits, especially as longer-term post-diagnostic support models are needed (Low et al., 2023). However, in this pilot, care partners indicated that receiving twelve weeks of intense intervention would be beneficial when they review content after program completion (D’Cunha et al., 2023).

Staff reported enjoyment and satisfaction in delivering an impactful, innovative multicomponent intervention for people with dementia and care partners. In addition to the unexpected benefits for staff, they described an increased confidence in running a dementia-specific program. Anticipated challenges such as participation fatigue and difficulty managing challenging situations proved unfounded. In the literature, Australian allied health professionals and university students report low confidence working with people with dementia, feel unsupported, and lack dementia-specific knowledge and education (Haydon et al., 2023; Lawler et al., 2021; Quick et al., 2022). While staff in the present study had varying levels of experience working with people with dementia, they were still unsure of these practicalities before commencing. Following the program, staff remained concerned about the absence of ongoing services for participants at the end of the program. However, group bonds formed among program participants and benefits emerging from social connections forged during the program were encouraging. To improve access to services such as the SPICE program, greater awareness of the benefits of non-pharmacological interventions during education and training of health professionals is needed to help change the narrative about the potential of dementia rehabilitation (Annear, 2018).

The study highlights that from the perspectives of staff, piloting a multicomponent dementia rehabilitation intervention is feasible and was not as burdensome as anticipated. Rehabilitation for people with dementia and care partners living in the community has attracted greater interest in recent years (Graff et al., 2023; Jeon et al., 2023; Thuesen & Graff, 2022), however, the key features of successful implementation remain unclear. In the current study, staff had varying levels of experience working with people with dementia, but all were optimistic the program could be successful. Key aspects of working with people with dementia in rehabilitation settings include the organisational culture, knowledge of staff, and personal values of healthcare (Hall et al., 2023). To deliver a successful rehabilitation intervention, a multidisciplinary team with strong leadership and motivated staff is essential to promote the function and independence of people with dementia and their care partners (O’Connor, Gresham, Poulos, Clemson, et al., 2020). The attitudes and motivations of the team are key factors in the success of dementia-specific rehabilitation interventions (Cations et al., 2020).

There remains significant stigma surrounding dementia (Alzheimer’s Disease International, 2024) and the rights of people with dementia to receive rehabilitation (Swaffer, 2021). While the World Health Organization recommends access to rehabilitation for people with dementia (Jeon et al., 2023), competing priorities and funding constraints often determine that dementia is not considered a priority in public health settings. However, interventions such as the SPICE program and comparable programs suggest the narrative may be starting to change (Casey et al., 2020, 2024; Clare et al., 2019; Maiden et al., 2024; Sondell et al., 2021; Teri et al., 2020). Given cost-related benefits have already been observed for the cognitive stimulation therapy (Knapp et al., 2022) and COPE (Rahja et al., 2020) components, further work is required to assess the economic benefits of combining evidence-based interventions in this population and to determine if they reduce risk of falls and avoidable hospitalisations.

The present findings highlight the importance of research on the implementation of complex interventions in health services. While a specific implementation science framework was not used to guide this exploratory work, the aim of the study and subsequent findings align with the Consolidated Framework for Implementation Research (CFIR). For example, the positive staff experience and high motivation levels can be seen through the “Inner Setting” domain which includes the organisational culture, engagement of leadership, and readiness for implementation. The staff involved in the SPICE program also placed value on their service providing access to dementia rehabilitation, and their confidence contributed to the success of the first group. The interaction between the staff perspectives and organisational context represents the importance of the “Process” factor of CFIR, as staff recognised the importance of evaluating the program and the opportunity it provides to reflect, refine and make improvements. Overall, the findings provide insights into the ways in which the program was implemented and achieved successful outcomes for the first group, with a view towards enhancing its sustainability as a novel dementia rehabilitation service. Future research on the evolution of multidisciplinary, multicomponent post-diagnostic interventions is needed to understand the key contextual factors impacting sustainability. A long-term evaluation of the SPICE program’s outcomes and a process evaluation are underway and will be critical to determine whether it is effective, scalable, and potentially adaptable to other health settings. Engaging stakeholders, including healthcare providers, people impacted by dementia, and policymakers, will be crucial to ensure ongoing support and funding for the program.

### Limitations

This study reports staff expectations and experiences in developing and implementing the first iteration of an innovative group-based multicomponent program for people with dementia and their care partners in a single location. The clinical and research team co-designed the intervention, with the motivation driven from within the service rather than being externally proposed or driven by senior management. Further, the study reports the experiences of staff following only one group of the intervention, thus limiting its generalisability. However, as the SPICE program is a holistic combination of evidence-based interventions for people with dementia and their care partners, the findings are important for health services that are considering starting a comprehensive post-diagnostic rehabilitation program in their setting. To increase the reliability and trustworthiness of the results, member checking was used, and multiple researchers were involved in independently coding the analysis. This involved researchers who had not directly observed the program to increase confidence in the findings.

### Conclusion

The findings from staff perspectives and experiences of the first SPICE program offer insights for health services considering implementing dementia rehabilitation programs. For successful implementation, there needs to be organisational and staff readiness, leadership, and adaptability. The expected challenges proved less concerning than anticipated, with none of the expected difficulties encountered in matters such as recruitment and retention or in staff capabilities in managing challenging situations. The positive outcomes highlight the benefits of healthcare providers and researchers working collaboratively towards sustainable initiatives which meet the needs of the community to enhance the quality and accessibility of care. Further research on future SPICE program groups and a process evaluation are ongoing which will contribute knowledge concerning multiple iterations of the program. Multicomponent rehabilitation programs have the potential to be adaptable to other settings, including community health and primary care, not-for-profit organisations, and residential care settings, warranting further research into their potential to delay progression of dementia and improve quality of life.

## Supplemental Material

Supplemental Material - Pilot of a multicomponent program for people with dementia and their care partners: Health services staff expectations, experiences and observations Supplemental Material for Pilot of a multicomponent program for people with dementia and their care partners: Health services staff expectations, experiences and observations by Nathan M D’Cunha, Georgina Chelberg, Helen Holloway, Lara Wiseman, Angie Fearon, Jane Kellett, Stephen Isbel, Kasia Bail, Ian Huang, Diane Gibson in Dementia

## ORCID iDs

Nathan M D’ Cunha https://orcid.org/0000-0002-4616-9931

Georgina Chelberg https://orcid.org/0000-0003-0305-8286

Helen Holloway https://orcid.org/0000-0003-3274-2095

Lara Wiseman https://orcid.org/0000-0001-8013-0523

Angie Fearon https://orcid.org/0000-0001-5055-3074

Jane Kellett https://orcid.org/0000-0003-0586-3203

Stephen Isbel https://orcid.org/0000-0001-5355-3205

Kasia Bail https://orcid.org/0000-0002-4797-0042

Ian Huang https://orcid.org/0009-0007-2292-2734

Diane Gibson https://orcid.org/0000-0001-7235-3719

## References

[bibr1-14713012251315527] Adeoye-Olatunde O. A. Olenik N. L. (2021). Research and scholarly methods: Semi-structured interviews. JACCP: Journal of the American College of Clinical Pharmacy, 4(10), 1358–1367. DOI: 10.1002/jac5.1441.

[bibr2-14713012251315527] Alzheimer's Disease International . (2024). World Alzheimer report 2024: Global changes in attitudes to dementia. https://www.alzint.org/u/World-Alzheimer-Report-2024.pdf.

[bibr3-14713012251315527] Annear M. J. (2018). Knowledge of dementia among the Australian health workforce: A national online survey. Journal of Applied Gerontology, 39(1), 62–73. DOI: 10.1177/0733464817752085. 29313420

[bibr4-14713012251315527] Australian Institute of Health and Welfare . (2023). Dementia in Australia. Australian Government. https://www.aihw.gov.au/reports/dementia/dementia-in-aus/contents/summary.

[bibr5-14713012251315527] Backhouse A. Ukoumunne O. C. Richards D. A. McCabe R. Watkins R. Dickens C. (2017). The effectiveness of community-based coordinating interventions in dementia care: A meta-analysis and subgroup analysis of intervention components. BMC Health Services Research, 17(1), 717. DOI: 10.1186/s12913-017-2677-2. 29132353 PMC5683245

[bibr6-14713012251315527] Casey D. Gallagher N. Devane D. Woods B. Murphy K. Smyth S. Newell J. Murphy A. W. Clarke C. Foley T. Timmons F. Dröes R.-M. O’Halloran M. Windle G. Irving Lupton K. Domegan C. O’Shea E. Dolan P. Doyle P. (2020). The feasibility of a comprehensive resilience-building psychosocial intervention (CREST) for people with dementia in the community: Protocol for a non-randomised feasibility study. Pilot and Feasibility Studies, 6(1), 177. DOI: 10.1186/s40814-020-00701-2. 33292667 PMC7667740

[bibr7-14713012251315527] Casey D. Smyth S. Doyle P. Gallagher N. O’Sullivan G. Murphy K. Dröes R.-M. Whelan B. (2024). An embedded qualitative study of the experiences of people with dementia, their caregivers and volunteer older adults who participated in the CREST resilience-building psychosocial intervention. BMC Geriatrics, 24(1), 780. DOI: 10.1186/s12877-024-05374-7. 39322962 PMC11423497

[bibr8-14713012251315527] Cations M. May N. Crotty M. Low L.-F. Clemson L. Whitehead C. McLoughlin J. Swaffer K. Laver K. E. (2020). Health professional perspectives on rehabilitation for people with dementia. The Gerontologist, 60(3), 503–512. DOI: 10.1093/geront/gnz007. 30759218

[bibr9-14713012251315527] Cations M. Radisic G. de la Perrelle L. Laver K. E. Shepherd K. Methorst F. Baldwin E. Maher-Norris D. Gibson J. Marsh E. Brown W. Palagyi J. Arndt P. M. Vladcoff K.-A. Sabja M. P. Caruana E. Tung J. Doljanin J. Anderson J. The Agents of Change Collaborative, G . (2019). Post-diagnostic allied health interventions for people with dementia in Australia: A spotlight on current practice. BMC Research Notes, 12(1), 559. DOI: 10.1186/s13104-019-4588-2. 31484587 PMC6727518

[bibr10-14713012251315527] Chung J. C. (2001). Empowering individuals with early dementia and their carers: An exploratory study in the Chinese context. American Joural of Alzheimers Disease & Other Dementias, 16(2), 85–88. DOI: 10.1177/153331750101600204. PMC10833943 11302076

[bibr11-14713012251315527] Clare L. Kudlicka A. Oyebode J. R. Jones R. W. Bayer A. Leroi I. Kopelman M. James I. A. Culverwell A. Pool J. Brand A. Henderson C. Hoare Z. Knapp M. Morgan-Trimmer S. Burns A. Corbett A. Whitaker R. Woods B. (2019). Goal-oriented cognitive rehabilitation for early-stage Alzheimer's and related dementias: The GREAT RCT. Health Technology Assessment, 23(10), 1–242. DOI: 10.3310/hta23100. PMC6441850 30879470

[bibr12-14713012251315527] Clemson L. Laver K. Rahja M. Culph J. Scanlan J. N. Day S. Comans T. Jeon Y.-H. Low L.-F. Crotty M. Kurrle S. Cations M. Piersol C. V. Gitlin L. N. (2021). Implementing a reablement intervention, “care of people with dementia in their environments (COPE)”: A hybrid implementation-effectiveness study. The Gerontologist, 61(6), 965–976. DOI: 10.1093/geront/gnaa105. 32803248

[bibr13-14713012251315527] D’Cunha N. M. Bennett M. Mitterfellner R. Brennan R. Wiseman L. Isbel S. Bail K. Barrett L. Rutherford K. Huang I. Gibson D. (2023). Preliminary findings of an active multicomponent lifestyle intervention for people with dementia and their carers: Mixed methods study. Health and Social Care in the Community, 2023(1), 5395080. DOI: 10.1155/2023/5395080.

[bibr14-14713012251315527] Department of Health and Aged Care . (2024). National dementia action plan 2024–2034. Australian Government. https://www.health.gov.au/sites/default/files/2024-12/national-dementia-action-plan-2024-2034.pdf.

[bibr15-14713012251315527] Desai R. Leung W. G. Fearn C. John A. Stott J. Spector A. (2024). Effectiveness of cognitive stimulation therapy (CST) for mild to moderate dementia: A systematic literature review and meta-analysis of randomised control trials using the original CST protocol. Ageing Research Reviews, 97, 102312. DOI: 10.1016/j.arr.2024.102312. 38636561

[bibr16-14713012251315527] Eaglestone G. Gkaintatzi E. Jiang H. Stoner C. Pacella R. McCrone P. (2023). Cost-Effectiveness of non-pharmacological interventions for mild cognitive impairment and dementia: A systematic review of economic evaluations and a review of reviews. Pharmacoeconomics Open, 7(6), 887–914. DOI: 10.1007/s41669-023-00440-z. 37747616 PMC10721583

[bibr17-14713012251315527] Gauthier S. Webster C. Servaes S. Morais J. Rosa-Neto P. (2022). World alzheimer report 2022: Life after diagnosis: Navigating treatment, care and support. https://www.alzint.org/u/World-Alzheimer-Report-2022.pdf.

[bibr18-14713012251315527] Gilbert E. Villa D. Riley G. A. (2023). A scoping review of psychosocial interventions to enhance the relationship of couples living with dementia. Dementia, 14713012231166474. DOI: 10.1177/14713012231166474. PMC10262342 37029512

[bibr19-14713012251315527] Gitlin L. N. Winter L. Dennis M. P. Hodgson N. Hauck W. W. (2010). Targeting and managing behavioral symptoms in individuals with dementia: A randomized trial of a nonpharmacological intervention. Journal of the American Geriatrics Society, 58(8), 1465–1474. DOI: 10.1111/j.1532-5415.2010.02971.x. 20662955 PMC2955191

[bibr20-14713012251315527] Graff L. Timm H. Thuesen J. (2023). Organizational narratives in rehabilitation-focused dementia care – negotiating identities, interventions and personhood. Dementia, 22(4), 709–726. DOI: 10.1177/14713012231161487. 36919376 PMC10088340

[bibr21-14713012251315527] Graneheim U. H. Lundman B. (2004). Qualitative content analysis in nursing research: Concepts, procedures and measures to achieve trustworthiness. Nurse Education Today, 24(2), 105–112. DOI: 10.1016/j.nedt.2003.10.001. 14769454

[bibr22-14713012251315527] Hall A. J. Manning F. Goodwin V. (2023). Qualitative study exploring health care professionals’ perceptions of providing rehabilitation for people with advanced dementia. BMJ Open, 13(7), e072432. DOI: 10.1136/bmjopen-2023-072432. PMC10391829 37524545

[bibr23-14713012251315527] Haydon H. M. Lotfaliany M. Jones C. Chelberg G. R. Horstmanshof L. Taylor M. Carey M. Snoswell C. L. Hicks R. Banbury A. (2023). Health literacy, dementia knowledge and perceived utility of digital health modalities among future health professionals. Australasian Journal oo Ageing, 42(2), 392–400. DOI: 10.1111/ajag.13149. 36334062

[bibr24-14713012251315527] Jeon Y.-H. Krein L. O’Connor C. M. C. Mowszowski L. Duffy S. Seeher K. Rauch A. (2023). A systematic review of quality dementia clinical guidelines for the development of WHO’s package of interventions for rehabilitation. The Gerontologist, 63(9), 1536–1555. DOI: 10.1093/geront/gnac105. 36043424 PMC10581378

[bibr25-14713012251315527] Kable A. Chenoweth L. Pond D. Hullick C. (2015). Health professional perspectives on systems failures in transitional care for patients with dementia and their carers: A qualitative descriptive study. BMC Health Services Research, 15, 567. DOI: 10.1186/s12913-015-1227-z. 26684210 PMC4683856

[bibr26-14713012251315527] Kishita N. Backhouse T. Mioshi E. (2019). Nonpharmacological interventions to improve depression, anxiety, and quality of life (QoL) in people with dementia: An overview of systematic reviews. Journal of Geriatric Psychiatry and Neurology, 33(1), 28–41. DOI: 10.1177/0891988719856690. 31203712

[bibr27-14713012251315527] Kjerstad E. Tuntland H. K. (2016). Reablement in community-dwelling older adults: A cost-effectiveness analysis alongside a randomized controlled trial. Health Economics Review, 6(1), 15. DOI: 10.1186/s13561-016-0092-8. 27165345 PMC4864744

[bibr28-14713012251315527] Knapp M. Bauer A. Wittenberg R. Comas-Herrera A. Cyhlarova E. Hu B. Jagger C. Kingston A. Patel A. Spector A. Wessel A. Wong G. (2022). What are the current and projected future cost and health-related quality of life implications of scaling up cognitive stimulation therapy? International Journal of Geriatriatrics & Psychiatry, 37(1). DOI: 10.1002/gps.5633. 34613622

[bibr29-14713012251315527] Laver K. Cumming R. G. Dyer S. M. Agar M. R. Anstey K. J. Beattie E. Brodaty H. Broe T. Clemson L. Crotty M. Dietz M. Draper B. M. Flicker L. Friel M. Heuzenroeder L. M. Koch S. Kurrle S. Nay R. Pond C. D. Yates M. W. (2016). Clinical practice guidelines for dementia in Australia. Medical Journal of Australia, 204(5), 191–193. DOI: 10.5694/mja15.01339. 26985848

[bibr30-14713012251315527] Laver K. Dyer S. Whitehead C. Clemson L. Crotty M. (2016). Interventions to delay functional decline in people with dementia: A systematic review of systematic reviews. BMJ Open, 6(4), e010767. DOI: 10.1136/bmjopen-2015-010767. PMC4854009 27121704

[bibr31-14713012251315527] Lawler K. Kitsos A. Bindoff A. D. Callisaya M. L. Eccleston C. E. A. Doherty K. V. (2021). Room for improvement: An online survey of allied health professionals’ dementia knowledge. Australasian Journal on Ageing, 40(2), 195–201. DOI: . 10.1111/ajag.12886. 33295077

[bibr32-14713012251315527] Livingston G. Huntley J. Liu K. Y. Costafreda S. G. Selbæk G. Alladi S. Ames D. Banerjee S. Burns A. Brayne C. Fox N. C. Ferri C. P. Gitlin L. N. Howard R. Kales H. C. Kivimäki M. Larson E. B. Nakasujja N. Rockwood K. Mukadam N. (2024). Dementia prevention, intervention, and care: 2024 report of the lancet standing commission. The Lancet, 404(10452), 572–628. DOI: 10.1016/S0140-6736(24)01296-0. 39096926

[bibr33-14713012251315527] Low L. F. Gresham M. Phillipson L. (2023). Further development needed: Models of post-diagnostic support for people with dementia. Current Opinion in Psychiatry, 36(2), 104–111. DOI: 10.1097/yco.0000000000000848. 36705009

[bibr34-14713012251315527] Maiden G. Kingsford A. Wang A. P. Tran-Nam A. R. Nelson J. (2024). Reimagining day rehabilitation for frailty and neurodegenerative conditions through the integrated rehabilitation and EnAblement program (iREAP). International Journal of Integrated Care. DOI: 10.5334/ijic.8066. PMC11414462 39308759

[bibr35-14713012251315527] McDermott O. Charlesworth G. Hogervorst E. Stoner C. Moniz-Cook E. Spector A. Csipke E. Orrell M. (2019). Psychosocial interventions for people with dementia: A synthesis of systematic reviews. Aging & Mental Health, 23(4), 393–403. DOI: 10.1080/13607863.2017.1423031. 29338323

[bibr36-14713012251315527] NHMRC National Institute for Dementia Research Special Interest Group in Rehabilitation Dementia . (2021). We need a model of health and aged care services that adequately supports Australians with dementia. Medical Journal of Australia, 214(2), 66–68. DOI: 10.5694/mja2.50911. 34414569

[bibr37-14713012251315527] O’Connor C. M. C. Gresham M. Poulos R. G. Clemson L. McGilton K. S. Cameron I. D. Hudson W. Radoslovich H. Jackman J. Poulos C. J. (2020). Understanding in the Australian aged care sector of reablement interventions for people living with dementia: A qualitative content analysis. BMC Health Services Research, 20(1), 140. DOI: 10.1186/s12913-020-4977-1. 32093699 PMC7041110

[bibr38-14713012251315527] O’Connor C. M. C. Gresham M. Poulos R. G. Hudson W. Jackman J. Clemson L. McGilton K. S. Cameron I. D. Radoslovich H. Poulos C. J. (2020). Translating reablement research for dementia practice: Development of a handbook using implementation science. Disability & Rehabilitation, 1–13. DOI: 10.1080/09638288.2020.1797910. 32772575

[bibr39-14713012251315527] Parkinson M. Carr S. M. Rushmer R. Abley C. (2017). Investigating what works to support family carers of people with dementia: A rapid realist review. Journal of Public Health, 39(4), e290–e301. DOI: 10.1093/pubmed/fdw100. 27679663 PMC5939885

[bibr40-14713012251315527] Pavković S. Goldberg L. R. Farrow M. Alty J. Abela M. Naismith S. Sachdev P. Low L.-F. (2023). Enhancing post-diagnostic care in Australian memory clinics: Health professionals’ insights into current practices, barriers and facilitators, and desirable support. Dementia, 23(1), 109–131. DOI: 10.1177/14713012231213419. 38116661 PMC10797845

[bibr41-14713012251315527] Pierse T. Keogh F. Challis D. O'Shea E. (2021). Resource allocation in dementia care: Comparing the views of people with dementia, carers and health and social care professionals under constrained and unconstrained budget scenarios. Aging & Mental Health, 1–9. DOI: 10.1080/13607863.2021.1889969. 33663288

[bibr42-14713012251315527] Quick S. M. Snowdon D. A. Lawler K. McGinley J. L. Soh S.-E. Callisaya M. L. (2022). Physical therapist and physical therapist student knowledge, confidence, attitudes, and beliefs about providing care for people with dementia: A mixed-methods systematic review. Physical Therapy, 102(5), pzac010. DOI: 10.1093/ptj/pzac010. 35157773 PMC9155993

[bibr43-14713012251315527] Quinn C. Pickett J. A. Litherland R. Morris R. G. Martyr A. Clare L. On behalf of the I. P. T. (2022). Living well with dementia: What is possible and how to promote it. International Journal of Geriatric Psychiatry, 37(1), 2. DOI: 10.1002/gps.5627. PMC9292841 34564897

[bibr44-14713012251315527] Rahja M. Nguyen K.-H. Laver K. Clemson L. Crotty M. Comans T. (2020). Implementing an evidence-based dementia care program in the Australian health context: A cost–benefit analysis. Health and Social Care in the Community, 28(6), 2013–2024. DOI: 10.1111/hsc.13013. 32431010

[bibr45-14713012251315527] Sondell A. Lampinen J. Conradsson M. Littbrand H. Englund U. Nilsson I. Lindelöf N. (2021). Experiences of community-dwelling older people with dementia participating in a person-centred multidimensional interdisciplinary rehabilitation program. BMC Geriatrics, 21(1), 341. DOI: 10.1186/s12877-021-02282-y. 34078266 PMC8173830

[bibr46-14713012251315527] Swaffer K. (2021). Chapter 1 - rehabilitation: A human right for everyone. In Low L.-F. Laver K. (Eds.), Dementia rehabilitation (pp. 1–13). Academic Press. DOI: 10.1016/B978-0-12-818685-5.00001-5.

[bibr47-14713012251315527] Teri L. Logsdon R. G. McCurry S. M. Pike K. C. McGough E. L. (2020). Translating an evidence-based multicomponent intervention for older adults with dementia and caregivers. Gerontologist, 60(3), 548–557. DOI: 10.1093/geront/gny122. PMC7117621 30304477

[bibr48-14713012251315527] Thuesen J. Graff L. (2022). Ageing, dementia and the future – ambivalent futurework in rehabilitation-focused dementia care. Dementia, 21(7), 2210–2230. DOI: 10.1177/14713012221117412. 35921632 PMC9483685

[bibr49-14713012251315527] Wheatley A. Bamford C. Brunskill G. Booi L. Dening K. H. Robinson L. on behalf of the PriDem study t. (2021). Implementing post-diagnostic support for people living with dementia in England: A qualitative study of barriers and strategies used to address these in practice. Age and Ageing, 50(6), 2230–2237. DOI: 10.1093/ageing/afab114. 34240114 PMC8675435

[bibr50-14713012251315527] World Health Organization . (2017). Global action plan on the public health response to dementia 2017-2025. https://www.who.int/publications/i/item/global-action-plan-on-the-public-health-response-to-dementia-2017---2025.

