# Supplementary material for: Pilot of a multicomponent program for people with dementia and their care partners: Health services staff expectations, experiences and observations
Source: Dementia (London). 2025 Jan 15;25(1):25–45. doi: 10.1177/14713012251315527 (PMC12701085; doi:10.1177/14713012251315527)
Supplement: Supplemental Material - Pilot of a multicomponent program for people with dementia and their care partners: Health services staff expectations, experiences and observations [file sj-pdf-1-dem-10.1177_14713012251315527.pdf]

SUPPLEMENTARY MATERIALS

Supplementary Figure 1. Pre- and post-program semi-structured interview questions for clinicians

**Pre-program questions**
Can you tell me a little bit about your professional self. For example:<br>• How long have you been a [profession]?<br>• Please tell us a little bit about your current role?<br>• What clinical areas have you worked in (e.g community, acute, mental health)?<br>• Do you have specific skills or experience in a specific area of your profession?<br>• What do you like about your profession and your current role?<br>Now, we would like to know a bit about your role in the development and/or implementation of the SPICE program.<br>• Overall what do you think about the SPICE program?<br>• Do you think it will be a good program? (why/why not)<br>• What elements of the program have you had input into or will be responsible for implementing?<br>• What do you think about the elements you will be involved in?<br>• What do you think are the best aspects of the program?<br>• What are the most challenging aspects of the program?<br>• Would you change anything in the program?<br>• How do you think the participants will find the program? (explain why)<br>• What do you anticipate will be the aspect the participants will improve the most in? (why)<br>• What aspects do you think the participants will find most challenging? (why)<br>• What do you think the carers will think of the program? (why)
**Post-program questions (Note: professional questions were asked if this was their first interview)**
• Overall what did you think about the SPICE program?<br>• Do you think it was a good program? Why/why not.<br>• Did the elements of the program you had input into work well? (why/why not)<br>• What do you think were the best aspects of the program?<br>• At the start of the program, you thought that […] would work really well. How did that turn out in the end?<br>• What are the most challenging aspects of the program?<br>• At the start of the program, you thought […] would be challenging. Did that turn out to be

correct?

- Would you change anything in the program? Why?
- How do you think the participants found the program?
- What areas did the participants improve in or get the most benefit out of, in your opinion? Did you expect this?
- What aspects did the participants find most challenging? Did you expect this?
- What do you think the carers thought of the program? Did you expect this?
- Could you think back and describe an event (or events) that stuck in your mind that best describes the program? (it could be a participant quote, a pleasing result, a discussion with a colleague, a carer reaction or just a personal reflection on the process).

Supplementary Table A: Benefits of the SPICE Program

Expectations before the initial intervention		
A1	Addressing a service gap	"I think it's excellent. And I think it's really needed and given that I am often the one giving the bad news to people with dementia, the idea of having a path forward rather than just going: '*There's your label. Sorry about it. Good luck.*' Yeah, that, that feels really nice…like it's the amount of services provided through this program - is kind of unprecedented in public health…"
A2		"If I'm honest, I can't believe that there isn't anything, that this is the only thing like it. At the moment, I think it makes so much sense to me to have all these things sit together in one service."
A3		"So, I think something like this, a program where we're actually looking at addressing quality of life, cognition, you know, wellbeing, your physical health is fantastic and a different option to medication, which isn't that effective."
A4	Benefits for staff	"So, talking about Canberra...most of the clinicians who are really keen to be involved, have probably experienced the level of distress and despair...of a care support provider, you know, or somebody living with dementia...that has been in a hospital system…they've had the person [care partner] talk about how difficult it is to care for the person with dementia...that's probably what I think is one of the things that drives people [staff]."
A5		"So, I think, to be able to hopefully, if the programme is effective, for me, as a clinician when I see a client progress, or have that 'aha' moment or feel supported, that's rewarding for me as well. So, I hope the SPICE programme would be rewarding for staff who are part of this development are going to feel rewarded as well."
A6		"I see this as a unique opportunity for developing a multidisciplinary team that's going to focus not only on the person with dementia but also their support network...[it is] unique and hopefully valued by everyone involved, not only the person with dementia and their carer, but also by the staff too."
A7	Benefits for care partners	"[To] be getting advice and strategies to help day to day. So, both the informal and formal, formal support of carers I think would be really valuable."
A8		"I also think one of the key things is supporting the carers and giving them strategies and helping them as a team, the person with dementia and their carer, [to] remain as safe and improve their quality of life and make sure that their stress levels are manageable, and behaviours are manageable."
A9		"…I think being able to have that all-encompassing approach will be really positive for them. And I hope as well as that, [the] connection of other people [carers] who are living with, you know, something else, something that you're living with…And hopefully, it might just improve some sustainability of people being able to continue in [the] lives that they want to lead at home, rather than ending up in crisis."

A10		"And I really like, that it's a program that is kind of valuing the skills and the knowledge of the carers, like the COPE program does. But also focusing on carer well-being, and kind of enhancing their skills and their quality of life and educating them."
A11	Benefits for people with dementia	"…I guess it's like any work that I do. If I didn't hold that belief and that as my underlying motivator, then we wouldn't be doing this. Like if we didn't think it was going to be helpful or effective, or supportive, we'd just be saying, well, let's just let dementia run its course and not intervene. So I have to hold on to any sort of intervention that I do that it's going to be helpful."
A12		"…the best aspects are around sort of having that people having sort of a holistic approach to, to the care of someone with dementia, so that care, the person with dementia and the carer, having that overall approach of the, you know, the exercise, the cognitive stimulation, the carers support, I think being able to have that all encompassing approach would be really positive for them."
A13		"I think, again, having that approach from all the different disciplines and making that cohesive rather than bits and pieces."
A14		"And the person with dementia yeah, I think I think just quality of life I think there's so much research evidence around you know, exercise and staying engaged and, and like the cognitive stimulation therapy. So I think the programme if there's one benefit, it would be improving the quality of life through physical and cognitive stimulation and engaging in activity."
A15		"I think that we'll find great improvements in their leg strength and their mobility, which should hopefully flow over into improvements objectable notable improvements in their quality of life, if they're able to do more than they were when they started."
A16		"…just so that kind of functional engagement in the world. [In a previous CST pilot] we saw that increase a fair bit, we saw that, well, their cognition on screening went up, which I wasn't really expecting at that point. I think their physical fitness will probably improve if they are not getting a lot of exercise currently. As I said, I don't know a lot about COPE, but I would think having tailored strategies in the home would also be really, really beneficial. In terms of getting people a bit more engaged in just everyday life, and not sort of sitting off in a corner isolated from things."
A17		"So from a cognitive perspective, things like I guess, general orientation. Just memory performance. You know, in terms of really discreet kind of measures, general engagement, though social engagement, I think, as well, so not just what we measure on the ACE."
A18		"I think it's really exciting because, yeah, like what the evidence shows it delays functional decline, it delays nursing home placement, and all of those things. I think the COPE programme is awesome. Yeah. And I think it's nice. It fits really well being kind of embedded within the SPICE project."

A19		"I hope they find it extremely supportive, informative, and fun. So I'm sort of thinking…the components of the programme…going home and doing OT work, and in looking at primary goals, and the CST can be really fun. And the gym stuff can be really fun. You know, like, I can envision that with, you know, music and dance and movement, and, you know, just making it fun…"
A20	Importance of program evaluation	"Something that we have to stick on our radar is how to make the outcomes that are provided for patients, or persons with dementia, [and] their carers, and how...we are able to communicate that in a way that is meaningful to people who are operating in a funding environment or model."
A21		"The other hurdle perhaps we've had is people's perception; health professionals, maybe the community…is rehab[ilitation], the right place for people with dementia? Like it's that conversation to have as well, Like, is it really rehab?…I think if we're looking at rehabbing, offering people the means to have the best quality of life that you can have, then certainly anybody's entitled to that."
Experiences and observations following the intervention		
A22	Addressing a service gap	"Just the kind of whole package and some of the recommendations that like the nutrition made fit really well with COPE, like simple things like changing to like a see-through fruit bowl so that people you know, making all see-through containers and making things accessible and stuff. So, it just was this really comprehensive program that fits well, together."
A23		"…I think all the features of the group of the whole program like the CST and the carer wellbeing and the exercise and everything, all worked really well together. But I think it works even better because it's the same group everytime from the start to the end."
A24		"The physiotherapist was able to adapt games to meet the different physical needs of the program participants which allowed participants with diverse abilities to engage in activities."<br>"…and they found ways to create exercise that was…fun. So, they didn't actually realise that they were actually engaged, particularly the people who didn't want to, they're engaging in some beneficial exercise."
A25		"I really liked the fact that I was involved in CST and COPE, because on the one hand, I got to, like, know that I knew stuff about the client that helps me kind of tailor the CST sessions to their like level. But then also, I suppose I got to observe the clients in like a different setting and that helped as well with the COPE program."
A26	Benefits for staff	"Oh it was just fantastic. To see the difference in probably all of the six clients, just the development in them. The communication, the rapport that was built amongst the clients, so I was mainly work[ing] with the clients not the carers, but I could see the rapport building on the carer side of things as well. It was very, very

		rewarding. Yeah".
A27		"I think earlier on in the group…the session when carers are coming in and saying '*I'm seeing changes to my person with dementia already'*, was probably a clinical highlight for me because it was a case of this is working already. It's only been three weeks. That was a great highlight. Seeing people in the class do things that I didn't expect."
A28		"…being able to interact, I guess with a group of people who they probably wouldn't normally have coming across the clinic…I think added to their learning."<br>"I think it was definitely good…to do the training through Dementia [Training] Australia that we had online. I think it was good."
A29	Benefits for carer partners	"I think when it comes to the COPE program yeah, for a lot of the participants [it] just increased engagement in like activities, and because the carers, had a better understanding of how to like how to engage them and how to support them, and like how to pitch it at the right level."
A30		"[F]or one of the participants who was wandering around at nighttime…they put in a strategy as a sign on the door at night that says, '*it's still nighttime'*... 'go back to sleep.' And their carer said that, you know, once they put [that] up, they slept through, they heard them up, but they didn't have to get up and then deal with a wandering person. And it's little things like that, that people don't necessarily, when you're in the middle of caring for somebody, you know, forest and trees, and you're tired, and you're overwhelmed, and you don't have those strategies to put in place."
A31		"[The] carer voiced on a number of occasions that you know, that she was trying very hard. And I think just the empathy shown there and the understanding that big, huge changes weren't necessarily the best way forward. That yeah, it needed to be achievable, and really tailored to his needs. The feedback that we got from that was, from the carer, was that they felt that they weren't judged, and that the advice was very achievable."
A32		"I still find it surprising that just that basic concept of self-compassion, you know, you can give someone just a few skills and it can really help them in terms of just them realising that, you know, that they can be flawed and that's fine."
A33		'…seeing the, watching the growth of the patients and their carers. And just hearing time and time again, the feedback and the positive feedback and the impact that it's made to the health well-being and quality of life of both groups of people…has been…really the highlight of the year from a work perspective."
A34		"[T]he carers have obviously found the program so effective that they have all said to us, '*if there's anything we can do or say or write or provide feedback for to help this you know, positive positively impacts other people then let us know and we'll happily*

		do that'."
A35	Benefits for people with dementia	"[participants] demonstrated carry over from one week to the next…so [they] were able to recognise the process…"
A36		"The first couple of sessions was such hard work. You know, teaching them how to do the exercise…But probably by the third or the fourth session, [whether] that was because of the carers as well as the clients, [I] didn't really know…that's what amazed me. The clients started to remember how to do them. They remembered….there were six stations so they would remember to go for number three, we go to number four. From number four, we go to number five, and by probably the last half of the exercise session, the last six sessions, we were just there telling them how great they were and go to the next one and we didn't have to give too much feedback at all to them".
A37		"I think there was some real gains, like people, like a lot of the carers did things outside of the strategies we gave them as well because they were learning and they, it was like they had a lightbulb moment. And yeah, like we had clients and people with dementia that you know, went back to feeding their dog. Or like one family, they bought a treadmill, or they started getting out music and stuff for them to listen to...[and] they did dancing."
A38		"Oh it was just fantastic. To see the difference[s] in probably all of the six clients [people with dementia], just the development in them. The communication, the rapport that was built amongst the clients."
A39		"He stood in the gym and was teaching me how to bounce it [AFL football] and so this was from somebody who, four weeks earlier, hardly spoke, [a] word was then already trying to teach me how to, to bounce a football and was laughing and just so engaged. And it was awesome. So, there's that. So, from somebody who just, you know, looked scared and afraid and you know, just couldn't engage in the program initially to somebody who was teaching me how to you know, bounce a footy that for me in just four weeks was a significant change."
A40	Social connection and relationships	"[T]hey were a cohesive group and they really supported one another and cared for one another."
A41		"[One of the carers told] us that it brought fun back into their relationship, which is something that they haven't had for so many years, which is not something we had put in a hypothesis of we think it's going to be really fun."
A42		"She didn't say I think it's made differences here and here. It was literally [that] it was something for us to do together that wasn't me caring for him or needing to look after him. We were able to do something entertaining and enjoyable together."
A43		"[The carers] took that upon themselves to create their own informal support network. And so, they would come and just sit and chat…they also talked about whilst the dedicated education

		and psychological and emotional skill-building sessions were great, they found the informal learnings that they got from each other through their own lived experience and…not only the learnings, but the emotional support they got through that was highly effective.”
A44		“The two ladies [with dementia] in the group got on extremely well. They like[d] to sit together and they often sort of linked arms and looked to each other for support and help especially one in particular. One of the clients was quite shy with other females and the other one who was quite out there. She sort of helped move her along and would encourage her in a funny sort of a way and where it would get the other client laughing. So, to see that each week, and we sat them together each week because the shy one felt safe with the outgoing one. So that was always nice just to see them. Yeah, they’d link arms, and they'd walk to the coffee together.”
A45		“…they did feel so supported and built up such reliable relationships and they did they do have their Whatsapp group or Facebook group that they’ve established.”
A46		“One of the clients, the last two visits came up to me…[H]e was very overwhelmed that it was coming to an end and one thing he said he said at both times was people listen[ed] to what I had to say. And he said ‘*You didn't brush us off. You listened. You made us laugh. Yeah, we had some good times. We had some bad times*.’ But to hear that. I thought that was amazing.”

Supplementary Table B: Challenges of the SPICE Program

Expectations before the initial intervention		
B1	Participant recruitment and retention	"I do think the program is busy. There are lots of components. And I, whilst I'm excited that we can put all of these together and provide that for people, I am a little bit worried about overload." "…and our neuropsych team tell us that whilst the project sounds really good and people are, will be potentially excited about it, actually being able to recruit the number of people who are happy to take a significant chunk out of their lives to attend the program will be difficult."
B2		"…that's a really long time to have people continuing to come in. And when you're dealing with an older population as well, who might have health issues going on, I think…could be really challenging." "…You've got dementia, you know the impact on fatigue and so forth afterwards… at home. I think it's a lot".
B3	Appropriate resources	"…the social work supervisor, who is not in our [SPICE] team, and I think her, her concern was like around the practicalities of it, you know, like all of that, in terms of the time commitment, and then having all our other work that we normally do as well, like, it's a big time commitment of clinicians."
B4		"We'll be putting in a lot for the size of our team. So, we have two and a half to three staff, servicing all of rehab [sic] and aged care for ACT. So, from our perspective, a clinician going twice a week to spend a couple of hours on this is a pretty big hit."
B5		"Because if it wasn't for her at the wheel and…whether someone else would be able to pick up that and keep it going, I would hope someone within our team would."
B6		"You do need a champion, you need a champion who is not going anywhere for a little bit. Because there is actually quite a high turnover in a lot of allied health."
B7	Staff capability to respond to challenging situations	"[A]n incident of some challenging behaviour that people don't feel like they've got the skills and knowledge to manage, that there's a significant fall, you know, for that for me, or it's… emotional breakdown of somebody, that would be a challenge.
B8		"There are smaller pragmatic challenges around matching the ability level of the people in the groups, personality clashes of people in the groups…criteria [that could] make it very difficult for somebody to engage in a group. So things like deafness, behavioural disinhibition?"
B9	Sustainability of the program	"For us just another challenge, though, that and something that we have to stick on our radar is, is how to make the outcomes that are provided for patients, or persons with dementia, their carers, and how we are able to communicate that in a way that is meaningful to people who are operate in a funding environment or model." "But for us that's another challenge for sustainability is again,

		being able to identify the value that comes from that program and being able to compete in a financial market where you can demonstrate the benefit…in line with, you know, the drivers for most health services, which are bed days…"
Experiences and observations following the intervention		
B10	Participant recruitment and retention	"…it totally flipped on its head, my expectation that if it's too long, it's too long for people to engage with and clearly they didn't think it was long enough. So it was just so different to our usual rehab cohort, not attending appointments and so forth. So that was a surprise for me."
B11		"…one of the things we need to wonder about in the setup was the bulk of activity, in terms of how it particularly sits with the COPE program…given the fact that [COPE itself is] quite an intensive program. Certainly, the feedback from all of the carers was that they found every component of the program beneficial. But the COPE overlay on top of getting to CST and the sessions at UCH, they found was really challenging…"
B12		"They found that the two together was too much to be able to implement [all] the strategies that were being suggested within the COPE program. Within the COPE program, the idea is that you, the OT suggests the strategies and the person tries to trial them for a week and then when the OT comes back again, they talk about what went well, what didn't work so well, to try and really help the carer hone in on some different modifications to those strategies…they just felt quite bad that they hadn't been able to put their strategies in place."
B13	Appropriate resources	"I think the two most like, time intensive weeks of the program of the COPE program and when I was trying to plan the CST sessions, I had like mandatory training and stuff and it was very busy. So, I would like to plan that a bit better next time."
B14		"We put this together through no additional funding…people kind of took on activity outside of existing caseload. The clinicians however, wanted to meet the need of people with dementia and their carer."<br>"I think now, it didn't, it doesn't feel like the time investment was as great as it felt like it was going to be on paper. So, for example, I think we were put on one OT this time, because we, we wanted to make sure that they were able to establish a new program. But we now feel like we would only need an additional half an OT to run two programs. You know, we would need a little bit the same, but also, again, I think, you know, after you run the first group, you get a little bit, you find your efficiencies."
B15	Staff capability to respond to challenging situations	"And actually like a lot of the clients didn't have like, it wasn't like the main focus, maybe these really big behavioural problems …So actually, it wasn't like I think that was the thing I was most worried about. But it didn't end up being the most challenging thing."

B16		"So you've got a lot of content you want to communicate to carers but at the same time, there's a lot of distress. So, you want to be validating as well. And managing, I guess individuals' distress levels, and group dynamics could be a little bit challenging at times."
B17		"Because you want to try and balance it out so that everyone, you know, has a fair say and contribution, but there was certainly some carers that were more dominant than others in terms of the group dynamic."
B18	Sustainability of the program	"So one of the things we are looking at doing is trying to find some funding so that we can implement two groups at a time, because again, I think the investment of time, we'll get some more resources, we'll get some more efficiency."
B19		"And then they also even said would happily pay for the program."<br>"…by the time people are saying you know, where we'd be happy to pay for it and obviously is again, feels like it's something they are finding incredibly valuable."

Supplementary Table C: Suggestions to improve the SPICE Program

Expectations prior to the intervention		
C1	Ongoing support as a follow-up to SPICE	"In an ideal world, if there was funding and staffing, then there would be some regular check-ins and support across after that program ends."
C2		"So after we stop, I suppose it's difficult because you don't want to just drop people, probably want to do some kind of follow up." "…a structured like sort of semi-structured phone call conversation about you know, how they're going, and I suppose the thing about that though, is that whatever follow up you do, if things are raised as an issue, then we need to know how we're going to direct them."
C3	Enhanced cultural inclusivity	"[I]n terms of...diversity issues, and people from different cultural backgrounds, and especially when you're running like education, and so forth, people from different cultural backgrounds might have a bit more, you know, difficulty picking up on some of those things."
Experiences and observations following the intervention		
C5	Ongoing support as a follow-up to SPICE	"…it's like having those like onwards referrals and links to like, ongoing things. After the program…just like the physiotherapist gave them, like, sort of exercise classes that they could go to after the program. Maybe doing that with some of the other components. The COPE program does really well at that like giving them the skills and generalising the strategies, but then a lot of people were asking about CST and whether there was anywhere else that implemented CST or like their, like there are CST manuals for implementing at home."
C6		"…but I just feel that they [carers] just got so much value out of the peer support, and of that regular, structured engagement with other people and I think it really difficult just for that to stop. And I know that some of them have been to other sort of carer groups before, but had not got a lot of value out of it, was the feedback to us…I think I'm not sure how we best support in that transition from you know, finishing the program, actually."
C7		"…yeah it's a shame that it ends because it fills such a need, but the same time resources make it really hard to continue. So I'm not sure."
C8	More individualised advice during the program	"Feedback from some people, some of the carers, that they would have liked to access to some more one on one social work, even if it was only a one or two sessions, just to have some dedicated time talking about their particular situation."
C9		"Because I think some people were hoping, like, the social workers or the psychologists would be able to like, answer all these very, like personal questions and address things, which it wasn't, it was definitely a group thing."

C10		"There were towards the end some clients talking about, you know significant concerns at home with, mobility aids being required in the near future for the person with dementia. But I didn't have a huge amount of capacity to really do any additional assessments...[or] to give them specific advice…"
C11	Clearer communication with care partners	"Setting expectations on the level of support that could be offered. We had a client that had like a long history of alcohol abuse and, and it caused a lot of distress to the person caring for him and was really challenging. And we did do some strategies around that. And I think it like it validated the carer. And they felt a lot more they felt more confident and, like less upset by the problem at the end, but we didn't like fix the problem."
C12		"On reflection I think we may eant to spend more time in that first session really asking [carer partners]…more what they want to get out of it. We did do that. But I think more time needs to be allocated to that space and finding out what they want out of it."
C13	Consideration of the program intensity	"So I think if I changed, it would be nice for it to align somehow so that you've maybe done some of the COPE sessions, like maybe the assessment sessions and then the CST started…"<br>"So I don't know if there's a way to adapt the timeline a little bit….Even do like two groups over a longer period of time."
C14		"…but already, you know, talking about whether or not there is a way to, you know, run the program over a bit of a longer time… maybe 24 weeks or 22 weeks, so there's that first component, and then we look at running the COPE program after that."
